# A case-control study of Metallothionein-1 expression in breast cancer and breast fibroadenoma

**DOI:** 10.1038/s41598-019-43565-0

**Published:** 2019-05-15

**Authors:** Fabiane Araújo Sampaio, Luana Mota Martins, Carla Solange de Melo Escorcio Dourado, Camila Maria Simplício Revoredo, Danylo Rafhael Costa-Silva, Victor Alves de Oliveira, Francisco Adelton Alves-Ribeiro, Benedito Borges da Silva

**Affiliations:** 1 Postgraduate Program, Northeast Biotechnology Network (RENORBIO), Federal University of Piauí, Teresina, Piaui 64000-020 Brazil; 2 Postgraduate Program in Health Sciences, Federal University of Piauí, Teresina, Piauí 64000-020 Brazil

**Keywords:** Prognostic markers, Breast cancer

## Abstract

The overexpression of Metallothionein-1 (MT-1) may play an important role in breast cancer; however, few studies have compared MT-1 expression between breast cancer and fibroadenoma. A cross-sectional controlled study was performed in 66 premenopausal women, aged 20–49 years, who had been histologically diagnosed with breast fibroadenoma or breast cancer. The patients were divided into two groups: group A, control (fibroadenoma, n = 36) and group B, study (breast cancer, n = 30). Immunohistochemistry was performed on tissue samples of fibroadenoma and breast cancer patients to evaluate the expression of metallothionein using an anti-MT-1 polyclonal antibody (rabbit polyclonal anti-metallothionein-Catalog Number biorbyt-orb11042) at a dilution of 1:100. The data were analyzed using NOVA (p < 0.05). Microscopic analysis showed a higher concentration of anti-MT-1-stained nuclei in breast cancer tissues than in fibroadenoma tissues. The mean proportion of cells with anti-MT-1-stained nuclei was 26.93% and 9.10%, respectively, in the study and control groups (p < 0.001). Histological grade 3 tumors showed a significantly higher MT-1 expression than hitological grade 1 (p < 0.05), while breast tumors negative for estrogen-, progesterone- and HER2- receptors had a significantly higher MT-1 expression than positive breast tumors positive for these parameters (p < 0.05). MT-1 protein in women of reproductive age was significantly higher in breast cancer than in fibroadenoma in this study. Furthermore, there was higher MT-1 immunoreactivity in more aggressive tumors.

## Introduction

Breast cancer is the most common malignancy that affects women in western countries and is the leading cause of cancer death among women worldwide^1,2^. In Brazil, 59,700 new breast cancer cases were estimated for the year 2018, with an estimated risk of 56.33 cases every 100 thousand women^3^. Although physical examination and mammography are important to ensure an early diagnosis of the disease and to reduce mortality, breast cancer is still frequently diagnosed at an advanced stage in Brazil. As a result, mortality rates are high even with current therapeutic strategies^4^.

Nevertheless, it has been suggested that more adequate therapeutic and prognostic strategies in breast cancer can be developed using protein biomarkers, such as metallothionein. This protein has the advantage of not only interfering with cell apoptosis and proliferation but also undergoing alterations well before the occurrence of clinical tumor alterations^5^ and thus may better guide treatment and prognostic strategies^6,7^. Metallothionein (MT) is a protein that has been widely studiedas a prognostic marker for breast cancer, since it promotes apoptosis, proliferation and differentiation of malignant tumor cells, making them more resistant to treatment^8–10^. Studies suggest that overexpression of metallothionein is associated with more aggressive breast tumors and more advanced-stage disease^11–14^.

Some studies have shown that increased expression of MT is associated with a higher proliferative potential in various types of cancer and a more rapid disease progression, as well as cellular resistance to chemotherapy and radiotherapy^15,16^. However, there is a paucity of studies evaluating the immunohistochemical expression of MT in breast cancer, and to the best of our knowledge, only one published study in the literature has compared the expression of MT-1 between women with breast cancer and women with fibroadenoma^14^. Furthermore, fibroadenoma is not classified as a tumor by some authors, but it is regarded as an aberration of normal development an involution (ANDI)^17^. Therefore, the conception of the current study was motivated by the paucity of studies that have compared MT-1 expression between fibroadenoma and breast cancer.

## Patients and Methods

### Study design

This study was approved by the Internal Review Board of the Federal University of Piauí (CAAE: 43447015.8.0000.5214). All patients signed a written informed consent form before study initiation. This is a cross-sectional case-control study involving patients seen at the Breast Clinic in Getúlio Vargas Hospital, Federal University of Piauí, Brazil from October 2014 to October 2016. Sample size was calculated and 71 patients were required for the study. However, due to technical problems there was a loss of 7% of the samples. As a result, the study included 66 premenopausal women distributed into 2 groups: A, control (fibroadenoma, n = 36) and B, study (cancer, n = 30). Premenopausal women with follicle-stimulating hormone (FSH) levels <30 mIU/ml, breast fibroadenoma or breast carcinoma, and no previous oncologic treatment were included in the study. Patients with fibroadenoma underwent histologic analysis of the tumors to confirm their benign status prior to immunohistochemistry.

### Immunohistochemistry for MT-1

Breast tissue samples fixed in buffered formalin were cut into 3 μm-thick sections. Sections were deparaffinized in xylol for 5 minutes, dehydrated in absolute ethanol, washed in buffered saline solution at pH 7.4 for 5 minutes and then treated for 5 minutes with 3% hydrogen peroxide (H_2_O_2_) in buffered solution to block endogenous peroxidase activity. For antigen retrieval, the slides were placed in racks containing 0.21% citric acid (pH 6.0) and heated in a microwave oven for 15 minutes at maximum power. The slides were cooled, and phosphate-buffered saline was added for a cooling period of 20 minutes. Tissue samples were incubated overnight at 4–8 °C with monoclonal antibody metallothionein. A polyclonal antibody against MT-1 (rabbit polyclonalanti-metallothionein – Catalog Number biorbyt-orb11042) was used at a dilution of 1:100.

The slides were rinsed with PBS-Tween and incubated with secondary antibody (anti-mouse BA 2000, Vector Laboratories, Burlingame, CA) for 30 minutes at room temperature. After being washed again with PBS-Tween, the slides were incubated with reagents from the ABC Elite detection system (PK 6100, Vector Laboratories) for 45 minutes at room temperature. The samples were rinsed once more with PBS-Tween and incubated with DAB (1.0 ml EnVision FLEX DAB for one drop of chromogen) and for 5 minutes. Finally, the slides were washed with distilled water, counterstained with hematoxylin, stained with ammoniacal solution, dehydrated with absolute ethanol, passed through Coplin jars containing xylol and mounted in Permount resin. Cells expressing metallothionein protein were identified by dark brown staining in the nucleus and cytoplasm.

### Quantitative method

Two observers, blinded to patient identity and previously unaware of any of the cases, performed quantification of protein expression using a light microscope (Eclipse E-400, optic microscope, Nikon, Tokyo, Japan) connected to a color video camera (CHC-370 N digital camera, Samsung, Seoul, South Korea), where image was captured and transmitted to a computer equipped with the Image Lab software program, version 2.3 (SOFTIUM Informatica Ltda, São Paulo, Brazil) for image analysis.

To determine metallothionein expression, we counted nuclei of stained cells under a microscope with a magnification of 400X. At least 500 cells of the breast epithelium were counted on each slide, in random fields, starting in the area of highest MT-1 concentration in the cell nucleus, using Processing Software and Image Analysis-Image Lab® (SOFTIUM Informatica Ltda, São Paulo, Brazil).

### Statistical analysis

Statistical analyses for this study were conducted using the software R, version 3.2.2. Data were expressed as frequencies, percentages, measures of central tendency and dispersion. The normality of the data was tested with the Kolmogorov-Smirnov test. The Levene test was used to verify data homogeneity. To compare more than two means between normal and homogenous data, we used Student’s t-test and ANOVA. Significant levels were set at p values ≤ 0.05.

## Results

**Figure 1:**
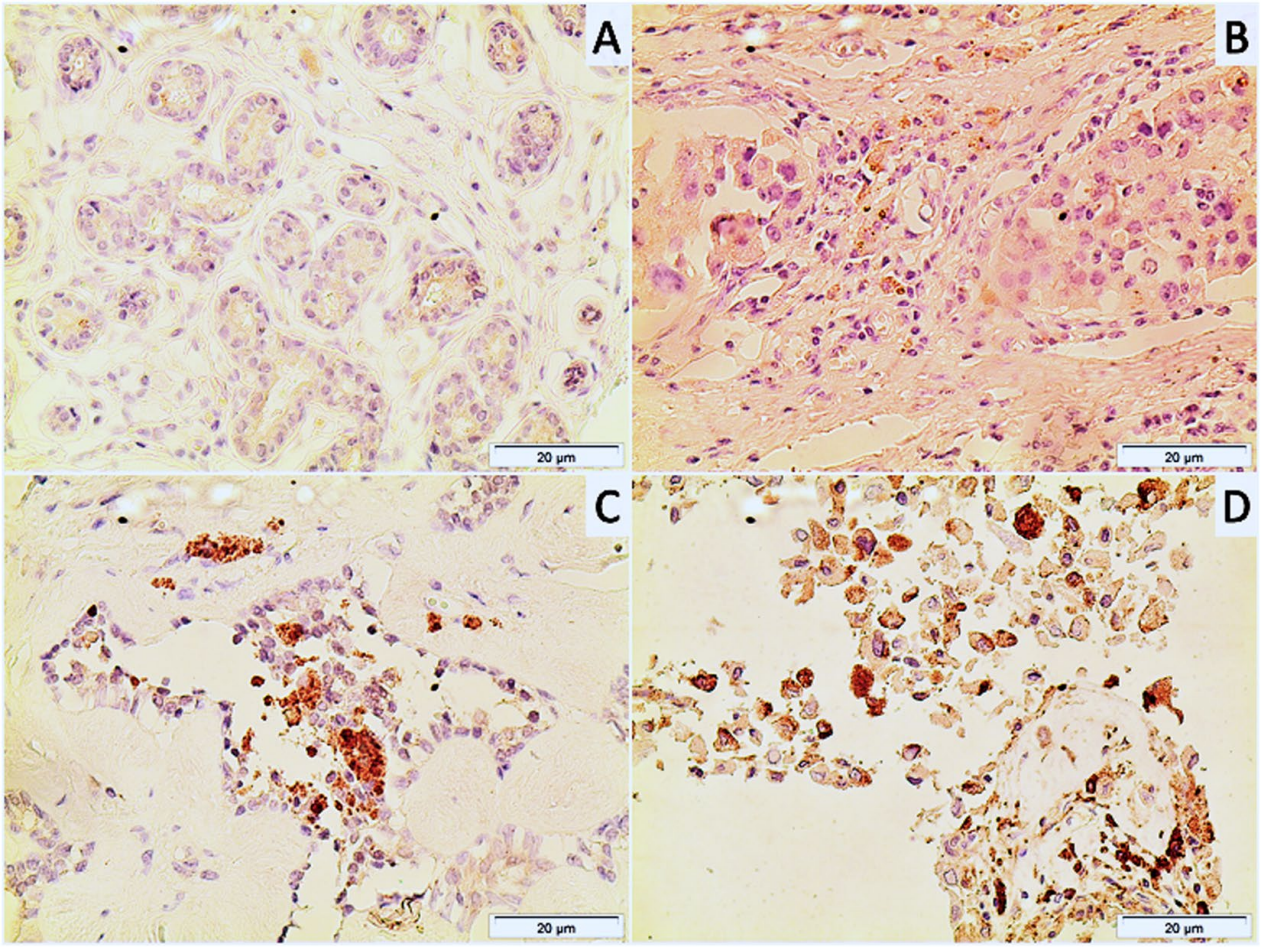
Photomicrographs of histological sections of breast fibroadenoma (**A**), grade 1breast cancer (**B**), grade 2 breast cancer (**C**) and grade 3 breast cancer (**D**). Note the higher concentration of MT-1-stained nuclei in breast cancer cells compared to fibroadenoma cells (original magnification X400).

Under optical microscopy, breast cancer cells had a higher concentration of nuclei stained with anti-MT-1 than fibroadenomas (Fig. 1). The characteristics of both groups were similar, except for age and waist circumference (Table 1).

**Table 1:** Characteristics of the patients in the study sample.

Characteristic	Group A (control) (n = 36) Mean ± DP	Group B (study) (n = 30) Mean ± DP	p
Age (y)	32.92 ± 9.46	40.37 ± 6.77*	0.0011
Menarche age (y)	12.86 ± 1.16	13.67 ± 1.79	0.0820
WC (cm)	79.40 ± 12.34	83.92 ± 8.92*	0.0480
BMI (kg/m²)	24.20 ± 4.61	25.70 ± 3.62	0.1540

*The mean of cases in group B was statistically significantly higher compared to group A.

Quantitative analysis showed mean percentages of nuclei stained with Metallothionein-1 per 500 breast epithelial cells in women at 9.10 ± 5.90 and 26.93 ± 15.87 in the control and study groups, respectively (Table 2).

**Table 2:** Mean percentage of Metallothionein nuclei per 500 cells in the control (A) and study (B) groups.

Group	n	Mean (%)	SD
Group A (Fibroadenoma)	36	9.10	5.90
Group B (Breast Cancer)	30	26.93*	15.87

*The percentage of cases with positive cells for Metallothionein in group B was statistically significantly higher compared to group A (p < 0.001).

**Figure 2:**
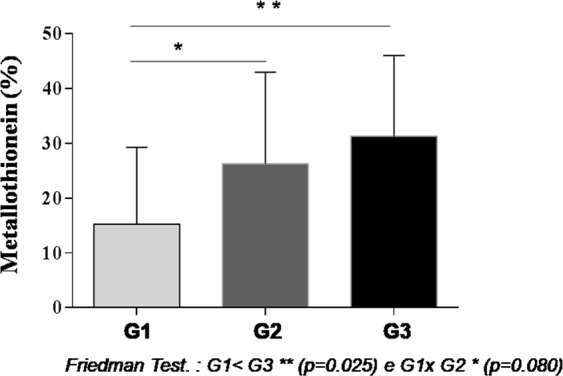
MT-1 expression in different histological grades of ductal invasive breast cancer.

Furthermore, Metallothionein-1 expression was statistically significant in histological grade 3 than in grade 1 tumors p < 0.05 (Fig. 2). MT-1 expression was statistically significant in breast cancers negative for HER2-, estrogen- and progesterone-receptors in comparison to tumors that were positive for these hormone receptors (p < 0.05), (Table 3).

**Table 3:** Metallothionein-1 mean**e**xpression in positive/negative tumors for ER, PR, and Her2.

Type of Receptors	Positive Mean ± SD	Negative Mean ± SD	p value*
ER	23.61 ± 15.97	30.24 ± 30.24	0.001
PR	23.12 ± 13.45	31.91 ± 17.88	0.001
HER2	25.97 ± 14.22	27.16 ± 16.53	0.007

*Teste ANOVA.

## Discussion

Metallothioneins participate in carcinogenesis by mechanisms promoting the development of tumor cells that are more resistant to chemotherapy or radiotherapy^15^. Elevated levels of this protein, with its antioxidant effect, can protect cancer cells against damage from free radicals. This protein has antiapoptotic and pro-proliferative effects, which support uncontrolled cellular growth in breast cancer^18–20^.

There is accumulating evidence that metallothionein is an immunohistochemical biomarker due to its elevated expression in myoepithelial cells of invasive breast carcinoma^12^. However, very few studies have attempted to elucidate the behavior of this protein in fibroadenoma, a benign tumor that does not increase the risk of developing breast cancer^13^, and this characteristic makes this condition an ideal control to determine the effect of metallothionein expression on prognosis in breast cancer.

In the current study, overexpression of Metallothionein-1 was observed in cells of breast cancer tissues relative to that in the cells of fibroadenoma tissues. Based on our literature search, only El Sharkawy and Farrag^14^ have investigated metallothionein expression in human breast fibroadenomas. According to those authors, higher MT-1 expression is related to more aggressive tumor behavior in ductal breast carcinoma. Other authors have provided additional confirmation that a higher nuclear expression of MT-1 is more frequently observed in carcinomas than in benign tumors^15,21^.

Furthermore, it is noteworthy that a significant difference was found between the mean age of breast cancer patients and breast fibroadenoma patients. Nevertheless, fibroadenomas are known to be more common in younger women, while breast cancer occurs more frequently in older women^22^. Waist circumference was larger in women with cancer, consistent with the literature, since premenopausal women with excess visceral fat have a higher risk of developing triple-negative breast cancer, which has a relatively worse prognosis^23^.

Agresti *et al*.^24^ observed that overweight premenopausal patients were at higher risk of developing triple-negative breast cancer than menopausal women. These findings suggest that obesity may play a role in the biological mechanisms underlying more aggressive types of breast cancer and the higher expression of metallothionein^25^.

Based on some authors, elevated MT expression blocks cellular apoptosis by sequestering zinc ions that stabilize p53, a gene that acts as a tumor suppressor by inducing apoptosis. Thus, MT enables the maintenance and integrity of the genome^26^. Recent studies indicate a strong relationship between p53 and MT, where overexpression of metallothionein is consistently associated with the presence of mutant p53, and in breast cancer, this relationship has been associated with a smaller number of apoptotic cells and a worse prognosis^27^.

Histologic tumor grade is associated with the immunoreactivity of metallothionein in breast cancer in addition to its anti-apoptotic effects. This discovery is consistent with previous observations in invasive breast carcinomas and *in situ* tumors reported by other researchers^10,12^. The current study showed a significant difference in MT-1 expression according to histologic tumor grade. Grade 3 tumors have a higher positivity for MT-1 protein, as well as HER2-overexpressing and negative hormonal receptors breast cancer. The association between these variables is relevant, indicating that metallothionein has a role in the differentiation, proliferation and progression of breast cancers. Proliferation is an important guide for the prognosis and therapy of malignant tumors^28,29^.

Therefore, studies have indicated that the expression of MT proteins in ductal breast cancer may represent an unfavorable prognostic index, since its highest expression is related to malignant cells with a higher histologic grade. Nevertheless, there is a need for further studies to obtain a better understanding of the behavior of metallothionein in tumorigenesis and to define the clinical significance of its expression in malignant and benign breast tumors.

### Ethical approval

The internal review board of the Federal University of Piauí approved this protocol. Informed consent was obtained from all individual participants included in the study. All the procedures performed in this study complied with current Brazilian laws and were in accordance with the ethical standards of the institutional and national research committees, as well as the 1964 Helsinki declaration and its later amendments.

## References

[1] Torre LA (2015). Global cancer statistics. CA Cancer J Clin.

[2] Ferlay J (2015). Cancer incidence and mortality worldwide: sources, methods and major patterns in GLOBOCAN 2012. Int J Cance.

[3] National Cancer Institute (INCA). Cancer estimates for 2018 in Brazil, http://www.inca.gov.br/estimate/2018.

[4] Anderson KN, Schwab RB, Martinez ME (2014). Reproductive risk factors and breast cancer subtypes: a review of the literature. Breast Cancer Res Treat.

[5] Dowset M (2006). A. Proliferation and apoptosis as markers of benefit in neoadjuvant endocrine therapy of breast cancer. Clin. Cancer Res.

[6] Duffy MJ, O’Donovan N, McDermott E, Crown J (2016). Validated biomarkers: The key to precision treatment in patients with breast cancer. The Breast.

[7] Ikink GJ (2018). Insertional mutagenesis in a HER2-positive breast cancer model reveals ERAS as a driver of cancer and therapy resistance. Oncogene.

[8] Fan LZ, Cherian MG (2002). Potential role of p53 on Metallothionein induction in human epithelial breast cancer cells. Br J Cancer.

[9] Lai Y, Yip GW, Bay BH (2011). Targeting metallothionein for prognosis and treatment of breast cancer. Recent Pat Anticancer Drug Discov.

[10] Bizón. A, Jedryczko K, Milnerowicz H (2017). The role of metallothionein in oncogenesis and cancer treatment. Postepy Hig Med Dosw (Online).

[11] Gomulkiewicz A (2016). Expression of metallothionein 3 in ductal breast cancer. Int J Oncol.

[12] Gallicchio LM, Flaws JA, Fowler BA, Ioffe OB (2005). Metallothionein expression in invasive and *in situ* breast carcinomas. Cancer Detect Prev.

[13] Shaik AN (2018). Breast fibroadenomas are not associated with increased breast cancer risk in an African American contemporary cohort of women with benign breast disease. Breast cancer Ressearch.

[14] El Sharkawy SL, Farrag ARH (2008). Mean nuclear area and metallothionein expression in ductal breast tumors: correlation with estrogen receptor status. Appl Immunohistochem Mol Morphol.

[15] Gumulec. J, Raudenska M, Adam V, Kizek R, Masarik M (2014). Metallothionein - immunohistochemical cancer biomarker: a meta-analysis. PLoS One.

[16] Wojtczak B (2017). Metallothionein isoform expression in benign and malignant thyroid lesions. Anticancer Res.

[17] Hughes LE, Mansel RE, Webster DJ (1987). Aberrations of normal development and involution (ANDI): a new perspective on pathogenesis and nomenclature of benign breast disorders. Lancet.

[18] Cherian MG, Jayasurya A, Bay BH (2003). Metallothioneins in human tumors and potential roles in carcinogenesis. Mutat Res.

[19] Jin R, Bay BH, Tan PH (2006). Metallothionein 1F mRNA expression correlates with histological grade in breast carcinoma. Breast Cancer Res Treat.

[20] Tai SK (2003). Differential expression of metallothionein 1 and 2 isoforms in breast cancer lines with different invasive potential: identification of a novel nonsilent metallothionein-1H mutant variant. Am J Pathol.

[21] Dincer Z, Jasani B, Haywood S, Mullins JE, Fuentealba IC (2001). Metallothionein expression in canine and feline mammary and melanotic tumours. J Comp Pathol.

[22] Sönmez K (2006). Surgical breast lesions in adolescent patients and a review of the literature. Acta Chir Belg.

[23] Fagherazzi G (2012). Hip circumference is associated with the risk of premenopausal ER−/PR− breast cancer. Int J Obes (Lond).

[24] Agresti R (2016). Association of adiposity, dysmetabolisms, and inflammation with aggressive breast cancer subtypes: a cross-sectional study. Breast Cancer Res Treat.

[25] Chen X (2010). Obesity and weight change in relation to breast cancer survival. Breast Cancer Research and Treatment.

[26] Ostrakhovitch EA, Olsson PE, Jiang S, Cherian MG (2006). Interaction of metallothionein with tumor suppressor p53 protein. FEBS letters..

[27] Ostrakhovitch EA, Song YP, Cherian MG (2016). Basal and copper-induced expression of metallothionein isoform 1, 2 and 3 genes in epithelial cancer cells: The role of tumor suppressor p53. J Trace Elem Med Biol.

[28] Gomulkiewicz A (2010). Correlation between Metallothionein (MT) expression and selected prognostic factors in ductal breast cancers. Folia Histochem Cytobiol.

[29] Sens MA, Somji S, Garrett SH, Beall CL, Sens DA (2001). Metallothionein isoform 3 overexpression is associated with breast cancers having a poor prognosis. Am J Pathol.

